# The Use of Rat and Mouse Models in Bariatric Surgery Experiments

**DOI:** 10.3389/fnut.2016.00025

**Published:** 2016-08-05

**Authors:** Thomas A. Lutz, Marco Bueter

**Affiliations:** ^1^Vetsuisse Faculty, Institute of Veterinary Physiology, University of Zurich, Zurich, Switzerland; ^2^Centre for Integrative Human Physiology, University of Zurich, Zurich, Switzerland; ^3^Department of Surgery, Division of Visceral and Transplant Surgery, University Hospital Zurich, Zurich, Switzerland

**Keywords:** RYGB, VSG, food intake, energy expenditure, rat, mouse, human

## Abstract

Animal models have been proven to be a crucial tool for investigating the physiological mechanisms underlying bariatric surgery in general and individual techniques in particular. By using a translational approach, most of these studies have been performed in rodents and have helped to understand how bariatric surgery may or may not work. However, data from studies using animal models should always be critically evaluated for their transferability to the human physiology. It is, therefore, the aim of this review to summarize both advantages and limitations of data generated by animal based experiments designed to investigate and understand the physiological mechanisms at the root of bariatric surgery.

## Introduction

Obesity and its related comorbidities have detrimental effects for the affected individual and pose a major challenge on public health systems worldwide. Despite the availability of a number of pharmacotherapies, the best treatment option leading to clinically relevant and maintained body weight loss is bariatric surgery (1–6). Bariatric surgery leads to a long-term reduction in body weight and in obesity-related morbidity and is currently the only treatment modality with a proven mortality benefit (4). Several techniques are currently employed. The gold-standard since many years is the Roux-en-Y gastric bypass (RYGB), followed by vertical sleeve gastrectomy (VSG) and, with decreasing numbers, adjustable gastric banding (AGB); the so-called mini-gastric bypass (MGB) has gained some popularity recently.

The treatment success of RYGB appears to be associated at least in part with changes in gastrointestinal hormones and bile acids that have been found to exert some role in the control of eating (7, 8). Despite the very different surgical approach, RYGB and VSG are associated with some extent with similar hormonal changes (9, 10). By contrast, reduced eating and weight loss after AGB is generally thought to result rather from mechanical restriction due to the reduced filling capacity of the stomach, although recent animal data suggest some role for gastrointestinal hormones, too (11). The MGB has been introduced ~20 years ago and has gained some popularity, in particular in non-academic surgical centers. However, surprisingly little research has been performed to study the underlying mechanisms that lead to body weight loss after MGB (12, 13).

Animal models have been proven to be a crucial tool for investigating the physiological mechanisms underlying bariatric surgery in general and individual techniques in particular. By using a translational approach, most of these studies have been performed in rodents and have helped to understand how bariatric surgery may or may not work. However, data from studies using animal models should always be critically evaluated for their transferability to the human physiology. It is, therefore, the aim of this review to summarize both advantages and limitations of data generated by animal-based experiments designed to investigate and understand the physiological mechanisms at the root of bariatric surgery.

## Role of Bariatric Surgery in Anti-Obesity Therapy

The number of non-surgical options to treat obesity are limited and the long-term success of dietary or life style interventions is minimal (2, 14). New drugs have recently been approved for obesity treatment, but long-term data are not available yet (1, 6). Insights into mechanisms of bariatric surgery, in particular RYGB and VSG, have opened up new treatment avenues against obesity. Among these, gut hormone-based strategies represent the most promising approach and are mainly focused on analogs of glucagon-like peptide-1 (GLP-1), such as liraglutide (Saxenda^®^) (15, 16). However, combinations of different hormones, such as amylin and leptin analogs, have also delivered remarkable results (17, 18).

However, due to an enormous discrepancy between the number of performed bariatric interventions, on the one hand, and the number of formally eligible patients worldwide, on the other hand [e.g., a number of ~0.5 Mio operations worldwide performed in 2013 (19) compares to the number of obese individuals of about 100 Mio in the USA alone (20–22)], it is obvious that the obesity epidemic cannot be successfully addressed by surgical means alone and that other non-surgical methods with a similar or superior efficacy and safety profile are urgently needed.

In this regard, research with animal models has significantly helped to elucidate some of the potential mechanisms underlying bariatric surgery. In comparison to human studies where investigating food intake is predominantly reliant on verbal report and dietary recall measures of patients, animal experiments allow the assessment of objective and unbiased data regarding postoperative changes of food intake. Furthermore, employment of genetic knockout models or specific antibodies directed against specific gut hormones or their receptors can only be performed in animals and have, thus, helped to differentiate between associative and causative relationships of proposed mechanisms of bariatric surgery. However, it needs to be emphasized that neither qualitative nor quantitative data generated in animals should be extrapolated to the human setting “one-to-one” and that animal studies unfold their additional value first of all in a translational experimental setting. In other words, it seems less relevant that weight loss rates are similar in rats and patients after RYGB, as long as the weight loss is mediated by similar physiological mechanisms in both settings.

Most animal research has been performed with RYGB and VSG interventions in rats or mice. Although the effect sizes of RYGB and VSG operations were found to show quantitative differences, it appeared that qualitative changes were remarkably robust between different studies. In other words, variables, such as surgical technique, pre-, peri-, and postoperative diet, baseline weight, and level of adiposity which all may affect the study outcome, were found to have a surprisingly little effect on the general information gain of these studies [discussed in Ref. (23, 24)].

## Animal Models of Bariatric Surgery Techniques

Although surgical methods to reduce body weight were first introduced more than 50 years ago and have been used in increasing frequency ever since, it is surprising that researchers only recently started to develop greater interest in post-bariatric physiological mechanisms. Koopmans and colleagues were among the first scientists who systematically used animal models to investigate the underlying mechanisms of bariatric surgery. These authors were able to demonstrate that a method called ileal transposition was not only effective in treating both genetic and hypothalamic lesion-induced obesity in rats, but also that the loss of body weight and body fat was associated with a reduction in eating. They further observed that ileal transposition caused hypertrophy of the small intestine and concluded that the early contact of the distal small intestine with undigested food and digestive enzymes may lead to an increased release of gastrointestinal hormones as one mediating factor (25–30).

Two other groups also made early contributions to the current literature of rodent models of bariatric surgery. Atkinson and Brent demonstrated that blood circulating factors seem to be critical for the reduction in eating after intestinal bypass operations in rats (31), and Meguid et al. were among the first to study altered brain signaling post-RYGB (32, 33). More recent studies built on these seminal experiments and the explanations how bariatric surgery and in particular RYGB reduce eating and body weight are still manifold. Finally, only a few groups published research with models of the AGB [e.g., Ref. (11)], and even less animal research has been done using the MGB technique (12).

## RYGB and VSG in Rats and Mice

The interest of the scientific community in post-bariatric physiology has grown exponentially over the last decade and many groups contributed significantly to the growing knowledge regarding the underlying mechanisms of bariatric surgery by using animal models of RYGB and VSG [e.g., Ref. (9, 10, 24, 34–55)]. Many of the reported effects showed striking similarities to what has been observed in RYGB or VSG patients.

For example, RYGB and VSG in rats and mice typically induced a rapid and sustained body weight loss which was mainly due to a reduction in body fat mass (40, 51, 56, 57). Post-surgical weight loss correlated to a large extent with reduced spontaneous eating, but an increase in energy expenditure may also play a role (51, 57, 58).

In addition, animal models also provided compelling arguments *against* traditional concepts such as intestinal malabsorption and mechanical restriction. There is nowadays a large body of evidence indicating that neither malabsorption nor restriction are the only mechanisms that exclusively explain the overall reduction in caloric intake and body weight after bariatric surgery (10, 51). In regard to caloric malabsorption, both animals and patients are able to digest and absorb ingested nutrients to a similar extent after RYGB than their respective controls. However, some reduction in fat digestibility has been described in rats and mice after RYGB when postoperatively exposed to a high-fat diet (35, 57), and a recent study indicated that intestinal glucose absorption may also be reduced after RYGB as a consequence of reduced sodium delivery and sodium-dependent glucose absorption in the proximal parts of the reconstructed small intestine (59). Thus, some role of maldigestion and malabsorption for body weight loss after RYGB needs to be considered in this scenario.

In regard to mechanical restriction, various studies have shown that RYGB- or VSG-operated rats and mice are indeed able to ingest larger quantities of food if they are metabolically challenged, e.g., subsequent to temporary food restriction after surgery (10, 24) or during lactation in female reproducing animals (60). Other arguments against a major impact of mechanical restriction on reduced eating after RYGB surgery include the observation that RYGB rats do not increase prandial drinking that might suggest an attempt to overcome mechanical constraint through food dilution with water. Furthermore, food intake after RYGB can be increased in rats and humans by somatostatin analogs that block the release of gastrointestinal hormones but which do not alter the mechanical situation post-RYGB (61, 62). In addition, differences in food intake and body weight are not related to the size of the gastro-jejunostomy in RYGB rats (63).

It needs to be emphasized that it was certainly wrong to explain the observed changes in eating behavior after bariatric surgery entirely and exclusively with mechanistic concepts, such as restriction and malabsorption, but it might be equally incorrect to neglect their impact. A typical finding after both RYGB and VSG operations is a change in meal pattern. Similar to RYGB patients, rats typically eat smaller meals after RYGB, which is partly compensated by an increase in meal frequency. In more detail, the size of nocturnal meals has been found to be markedly reduced post-RYGB, while the size of diurnal meals was actually increased compared to sham-operated control animals (23, 42, 51, 64). Furthermore, even if RYGB or VSG rats are metabolically challenged leading to high levels of total food intake (e.g., temporary food restriction; pregnancy and lactation), the rats do not seem to increase their meal size (10, 24, 60). Finally, a recent study showed that increased intake induced by antagonizing melanocortin-4 (MC4) receptors in the hypothalamus was entirely dependent on an increase in meal number, but not meal size (65). In summary, the available data indicate that RYGB (or VSG) rats may be mechanically restricted in a sense that the amount of food that can be eaten in a single meal is limited, but that rats are able to adapt to specific metabolic situations by increasing meal frequency.

## Mechanistic Studies to Explain Reduced Eating and Weight Loss after RYGB and VSG

Animal experiments provide valuable insight into the mechanisms that are at play after bariatric surgery and that lead not only to a reduction of body weight but also to long-term maintenance of the lower body weight. The available data indicate that RYGB- or VSG-operated individuals develop a new set point of their body weight that is defended even if challenged by certain experimental conditions (e.g., temporary food restriction or forced overfeeding).

Leptin may play an important role in the control of this set point defense because leptin-deficient ob/ob mice do not exhibit the same benefits in body weight and metabolic control compared to control animals unless leptin is replaced (66). Thus, the bariatric surgery procedure itself may be most critical for the extent of weight loss until the newly defended body weight may be reached. This may also indicate that further temporary manipulations (e.g., additional calorie restriction, alterations in food composition) may not necessarily result in additional long-term benefits.

The latter point is also important in a different context. Various studies showed that RYGB and VSG may lead to an alteration in food preference with rats and patients choosing to eat less high-fat and sugary foods in favor of less energy dense alternatives when offered a choice (42, 53, 57, 67–69). However, a recent review of the human literature found that reported changes in dietary macronutrients after RYGB were modest and only transient in nature (70). Although alterations in dietary selection could conceivably contribute to improved glycemia and body weight after RYGB and VSG surgery, it remains unclear whether they represent an essential contributor to these beneficial effects after surgery or not. Based on the findings reported in the previous paragraph, this may actually not be the case, with the respective consequences for dietary counseling.

Roux-en-Y gastric bypass and VSG lead to characteristic changes in the concentration of gut hormones and bile acids (8, 41, 71–73), which is a robust phenomenon consistently reported in basically all published studies. Nevertheless it needs to be stated that most attempts to establish a causal role of gut hormones and bile acids for the post-bariatric outcome have failed so far. For example, data obtained from GLP-1 receptor knockout animals or using GLP-1 receptor antagonists are negative in that RYGB- or VSG-induced effects did not differ from wildtype control animals (43, 74–76). Thus, while changes in gut hormones alone cannot explain the RYGB- or VSG-induced effects, it appears that rather a combination of multiple physiological alterations and interactions are at play. These include elevations in basal or postprandial concentrations of many gut hormones [GLP-1, cholecystokinin (CCK), amylin, peptide YY (PYY), etc.], increased levels, and altered composition of bile acids, as well as alterations in the diversity and composition of gut microbiota after bariatric surgery (7, 35, 41, 71, 76–78).

The large number of studies describing changes in the periphery after bariatric surgery contrasts with the remarkable paucity of data addressing changes in central nervous system function that may explain the effects of bariatric surgery. The most in-depth studies described the potential contribution of MC4 receptor signaling that is an important center point for the control of energy balance in general. The published data indicate that there may be a species difference in the relevance of MC4 signaling, because VSG effects were still present in MC4-deficient rats (46). By contrast, RYGB-induced changes differed between MC4-deficient mice and respective controls, and there appeared to be a gene dosage effect (34, 44, 79). The latter may also explain why RYGB or VSG patients with mutations in the MC4 gene typically still respond to bariatric surgery because they do not correspond to a full receptor knockout. Interestingly, some rare mutations in the MC4 were associated with a bigger weight loss and a faster resolution of diabetes post-surgery (80) [but see Ref. (81)]. Recent data indicated that RYGB also changes signaling in feeding areas of the caudal hindbrain in RYGB rats (82) but no equivalent human data are currently available.

Future experiments need to be designed to mimic specific aspects of bariatric surgery and to define the causal role of specific mechanisms for the beneficial effects of bariatric surgery. Manipulations may, e.g., include the local infusion of nutrients in specific gut segments, manipulations of nutrient contact with the gut mucosa, diversion of pancreatic juices and bile acids, and perhaps also the transposition of specific gut segments, similar to the procedure that Koopmans and colleagues had used more than 30 years ago (25, 29, 30).

## Comparison of Animal Models in Bariatric Surgery Research with the Clinical Situation in Humans

The validity of animal model data depends on the similarities in phenomena and mechanisms between humans and animals. For the most part, it seems safe to say that the similarities largely outnumber potential differences that may be more quantitative than qualitative.

The key effect of bariatric surgery seems to be a loss of excessive fat mass by resetting the system of body weight control in both animals and humans (10, 56, 66). In humans, loss of excessive body weight is typically more pronounced in more obese patients (83). On the other hand, diabetic patients with a body mass index between 22 and 35 lose on average ~20% of their total weight after RYGB (84), which is markedly less than the typical weight loss in heavier patients. Interestingly, a similar phenomenon is seen in animals where post-RYGB body weight loss also seems to correlate with the degree of preoperative obesity. Obese OLETF rats, i.e., rats that are obese because they overeat due to the lack of functional CCK1 receptors, lost markedly more weight compared to their lean LETO controls after RYGB (49), and recent studies in mice with different degrees of obesity corroborated these findings (56).

One aspect that differs between rodents and humans is the difference in weight growth curves; i.e., in contrast to obese humans, where the “control condition” typically refers to a stable body weight, control groups of rats or mice often gain weight over the observation period of a study. Here, the effect of bariatric surgery may be a prevention of this weight gain rather than an absolute weight loss. However, animal models allow the detailed study of major components contributing to the body weight loss in standardized and reproducible conditions, i.e., reduced caloric intake, increased energy expenditure, or reduced energy availability from ingested nutrients. By contrast, data on food selection and intake in humans rely in most cases on self-reported food intake that is vulnerable to inaccuracy for several reasons (70).

A further advantage of animal models is the use of specific control groups for reduced caloric intake or weight loss in respect to the metabolic consequences of bariatric surgery; in other words, pair fed or body weight matched controls, or controls for specific metabolic situations allow to distinguish bariatric surgery effects that are specific to the surgical manipulations, such as the anatomical re-arrangement of the small bowel anatomy versus effects that are rather a consequence of the induced weight loss (23, 24, 50, 51, 71, 85).

As discussed, body weight loss after bariatric surgery is mainly due to reduced energy intake and changes in meal patterns (42, 51, 64), and alterations in food choice and taste preference may also play a role (38, 42, 47, 53, 67, 69, 86). Further important similarities between animal models and humans undergoing bariatric surgery include changes in the postoperative profile of gut hormones and bile acids, but also the metabolic beneficial effects of bariatric surgery. The latter comprise, e.g., rapid improvements of insulin sensitivity, insulin secretory capacity, and cardiovascular function [e.g., Ref. (36, 45, 50, 71, 87–99)]. Similar to the effects on energy balance, a large number of follow-up experiments were performed to study the potential mechanisms underlying the metabolic effects of bariatric surgery. This included the manipulation of hormone systems or signaling cascades, and some studies clearly indicated that changes induced by bariatric surgery, e.g., elevated GLP-1 levels, do contribute to post-surgery metabolic effects in rats and humans (71, 93). However, other studies revealed rather disappointing results in a sense that blockade of GLP-1 signaling was often not able to offset the effects of bariatric surgery (75, 76).

Another contributing factor to weight loss after bariatric in humans and animal models may be the change in complexity and diversity in the gut microbiota. RYGB and VSG alter the composition of the gut microbiota and transplantation studies indicate that these alterations may also play a causal role in the improved metabolic status after bariatric surgery (35, 73, 100). For example, by colonizing germ-free mice with stools from the patients, Tremaroli et al. demonstrated that the surgically altered microbiota promoted reduced fat deposition in recipient mice. Mice also had a lower respiratory quotient, indicating decreased utilization of carbohydrates as fuel, suggesting that the gut microbiota may play a direct role in the reduction of adiposity observed after bariatric surgery (101).

Finally, not only the beneficial but also the negative consequences of bariatric surgery seem to be recapitulated in animal models, similar to what is seen in human patients. To give just three examples, RYGB causes a demineralization of the skeletal system potentially leading to an increased risk in bone fractures (85, 102–104). The underlying reasons for this effect are not clear, but own results indicate that a more acidotic status post-RYGB leading to increased calcium release from the bone may play a role. Second, RYGB may increase the risk for excessive alcohol intake in patients (105, 106) – a behavior which was also found in rats that did not prefer alcohol before the surgical intervention. Third, even though the metabolic status improves markedly in most diabetic patients after RYGB, some patients were found to have large fluctuations of their blood glucose concentration after RYGB surgery, especially in the periprandial phase paralleled by prolonged episodes of hypoglycemia. Similar findings have been reported in rodent RYGB models. The reason for these larger than normal glycemic fluctuations is not entirely clear, but may be linked to increased secretion of GLP-1 leading to a strong increase of insulin release which then may require the compensatory release of counterregulatory hormones. In other words, despite the markedly improved general metabolic status, the fine tuning of glucose control may not be achieved by RYGB (50, 107–111).

## Notes of Caution on the Use of Animal Models in Bariatric Surgery Research

Not all findings reported in rodent bariatric surgery models find their direct equivalent in the clinical situation, or vice versa, but some of the differences among species may be more quantitative than qualitative. Four examples will be discussed here.

First, the weight loss of RYGB and VSG in rats and mice is due to a reduction in eating and an increase of energy expenditure (respectively, the prevention of its decrease in weight-reduced animals). The relative importance of the energy expenditure component seems to be bigger in mice than rats; in fact, in some mouse studies, increased energy expenditure appeared to explain most of the surgery induced weight loss because food intake in RYGB-operated mice was higher than that in sham-operated controls (40, 51). By contrast, only some studies in humans report an increase in total energy expenditure. However, similar to the increased diet-induced thermogenesis that has been reported in rats, postprandial energy expenditure also seems to increase in some RYGB patients (55, 112–115).

The reason for the real or apparent species differences in respect to energy expenditure after RYGB is not clear but it may be more a general phenomenon of biology and physiology rather than a specific finding after bariatric surgery. The control of energy balance via energy expenditure may be much more efficient in mice with their large body surface to body mass ratio; in humans, this ratio is opposite, and this may be reflected in the more important control of energy balance via energy intake. Rats may be between both extremes, and this may explain why both energy intake and energy expenditure are typically affected by bariatric surgery in rats.

Second, bariatric surgery and in particular RYGB and VSG also lead to changes in food selection, and some reports claim that consumption of high-fat and sweet foods decreases post-RYGB (70, 116, 117). Similar findings have been reported in rats and mice because they chose to ingest lower amounts of high-fat or high-sugar diets than sham-operated controls. This decrease in intake is progressive and is reminiscent of a learning process (conditioned avoidance) (40, 42, 53, 57, 68). Interestingly, the decrease in sugar intake may also be due to an altered taste sensitivity because RYGB has been shown to lower the sucrose detection threshold in patients after RYGB (67) [see also Ref. (117, 118)]. Furthermore, brief access tests in rodents often did not indicate reduced avidity for sucrose or high fat and rats’ voluntary work for sucrose or lipid solutions is not decreased (119–122). Nonetheless, whether the findings in animal models can be directly translated into the human situation is not clear, and only few objectively assessed data in humans are available. Changes in macronutrient intake in rats seem to be a long-term effect, while lasting changes in relative macronutrient intake in humans have typically not been observed. In other words, it is not clear whether the proportion of fat in the diet of post-RYGB individuals is decreased over extended periods of time, and it is also not clear if and for how long changes in diet composition contribute to reduced energy intake and weight loss in RYGB-operated patients.

Third, a typical finding in RYGB-operated rats is a massive hypertrophy of the intestinal wall in the Roux limb and to a lesser extent in the common channel of the RYGB reconstruction (51, 77, 123–125). The hypertrophic small intestinal epithelium may contribute to the increase in total energy expenditure in rats, and it may contribute to sufficient nutrient digestibility and absorption despite the altered gut anatomy. Whether the human gut hypertrophies to a similar extent in RYGB patients is still a matter of debate and only few well-controlled studies have been performed. One recent study, however, clearly indicated that the small intestine in RYGB patients showed a clear hypertrophic response (126). Furthermore, anecdotal evidence also reports that gut hypertrophy may also occur in RYGB patients; in a rather dramatic recent case, a short common channel was associated with massive mucosal hypertrophy eventually leading to a functional ileus (personal observation, Marco Bueter). The general extent and underlying mechanisms of gut hypertrophy post-bariatric surgery will need to be defined in further well-controlled clinical studies.

Finally, potential influences of anatomical differences between rodents (or other animal models) and humans need to be considered even though evidence for an important impact of these differences on study outcome is limited. The gastrointestinal anatomy differs between humans and rodents in some aspects. Mice, e.g., have an extensive portion of their stomach covered by cutaneous mucosa (called “forestomach” by some), and the proventriculus in rats also has no human equivalent. Furthermore, mice but not rats have a gall bladder. Despite that, delivery of bile into the proximal small intestine is also dependent on CCK in rats and no principal difference seems to exist between mice, rats, or humans in respect to the elevation of circulating bile acids after RYGB and VSG (8, 59, 71, 73, 88, 127–130). Of note, there are also some differences in the bile acid profile post-RYGB in humans compared to rat or pig RYGB models. Some bile acid species, such as the free bile acids cholic acid, chenodeoxycholic acid, and deoxycholic acid were similarly increased, but glyco-conjugated bile acid species concentrations depended on the animal model, and no global increase in tauro-conjugated species was observed. These differences may be relevant because different bile acid species have different affinity and efficacy at the various bile acid receptors (130).

## Models of Bariatric Operations Other than RYGB and SG

### Mini-Gastric Bypass

Knowledge from previous experiments can now be used for the optimized design to study mechanisms of more recently introduced bariatric surgery procedures. The so-called MGB has gained some interest because it has the reputation to be an easier version of the classical RYGB. However, very little information about possible mechanisms of action is available. More importantly, data about the long-term effects and potential negative consequences are not available so far. Interestingly, this technique has been reported to lead to an increase rather than a decrease in eating. Energy expenditure has not been studied in detail after MGB, but it is generally assumed that maldigestion and malabsorption may be important components in the weight lowering effect of MGB (12, 13, 131, 132). If the latter were the case, the mechanisms of action would clearly differ between RYGB and MGB and would put a note of caution on the use of the MGB due to the potential of developing deficiencies in essential nutrients.

### Biliopancreatic Diversion

The biliopancreatic diversion (BPD) introduced and developed by Scopinaro consists of a partial gastrectomy with a Roux-en-Y gastro-jejunostomy forming an alimentary limb and a duodeojejunal biliopancretic limb anastomosed to the distal ileum. The operation leads to significant weight loss with normal absorption of bile salts, water and electrolytes (133). This operation is generally performed in much lower numbers than RYGB and SG and has its main indication in severely and mega-obese patients (19). Rat models of BPD revealed that serum protein, cholesterol, and triglycerides fell by 25–40% postoperatively (134), while the procedure was associated with intestinal hypertrophy and with increased GLP-1, GLP-2, and PYY levels (135).

### Biliopancreatic Diversion with Duodenal Switch

To preserve physiologic gastric emptying and to prevent anastomotic ulcer after BPD by decreasing the effects of alkaline biliary reflux, Hess developed a modified BPD procedure with the alimentary limb being directly anastomosed to the post-pyloric duodenum (136). This operation is today known as BPD with duodenal switch (BPD-DS) and also includes a VSG, before the use of VSG as a stand-alone procedure (137). The BPD-DS is considered by some as the most efficient surgery in treating obesity and T2DM, but the rate of early complications is higher and it might also be associated with a higher perioperative mortality (138); for this reason, the BPD-DS is not extensively performed worldwide (19). BPD-DS operations in rats showed that the procedure is associated with an increased fecal energy loss as well as a (compensatory) intestinal hypertrophy with elevated levels of fasting and postprandial plasma GLP-1 and PYY (139), while there is a reduced expression of thermogenic genes in the interscapular brown adipose tissue (140).

## Outlook and Conclusion

Overall, rat and mouse experiments in bariatric surgery have been proven to be an important and relevant research tool that has led and will lead to important findings translatable into the clinical situation. Some differences have been identified, but careful experimental designs still allow clinically relevant conclusions. More studies are needed that directly compare effects and consequences of bariatric surgery procedures across species. This includes the assessment of similar parameters pre- and post-bariatric surgery in human patients and animal models, but also similarly designed experiments that yield mechanistic information in all relevant species. Only few examples are available in the literature [e.g., Ref. (67, 70, 121, 122, 129, 130)]. Nonetheless, without animal models, our knowledge on how bariatric surgery works (or may not work!) would be very limited and the vast literature that is available indicates that most animal models seem to recapitulate remarkably well the findings in humans. Future research in animal models of bariatric surgery will most likely include the more frequent use of larger animal models, e.g., minipigs or dogs (129, 141, 142). Larger animals offer significant advantages compared to rats and mice; e.g., larger blood volumes can be collected over extended periods of time, and specific interventions in defined parts of the gastrointestinal tract may be easier to perform in larger animal models. Furthermore, specific aspects of energy expenditure may be more similar to humans in larger animals compared to small animals, in particular the mouse.

## Author Contributions

TL wrote paper, performed literature research, and produced final version. MB revised paper, performed literature research, and edited text.

## Conflict of Interest Statement

The authors declare that the research was conducted in the absence of any commercial or financial relationships that could be construed as a potential conflict of interest.
